# Supernumerary phantom limb in a patient with basal ganglia hemorrhage - a case report and review of the literature

**DOI:** 10.1186/s12883-017-0962-7

**Published:** 2017-09-08

**Authors:** Hang-Rai Kim, Jee-young Han, Young Ho Park, Beom Joon Kim, Wookjin Yang, SangYun Kim

**Affiliations:** 1 Clinical Neuroscience Center, Seoul National University Bundang Hospital, 82, Gumi-ro 173, Beon-gil, Bundang-gu, Seongnam-si, Gyeonggi-do 463-707 Republic of Korea; 2 Department of Neurology, Seoul National University College of Medicine, Seoul, Republic of Korea

**Keywords:** Supernumerary phantom limb, Basal ganglia hemorrhage, Intentional movement

## Abstract

**Background:**

Supernumerary phantom limb (SPL) is a rare neurologic phenomenon, in which a patient misperceives an extra limb in addition to the original set of limbs. We report a case of SPL in a patient with a right basal ganglia hemorrhage and review the previous literature about this peculiar phenomenon.

**Case presentation:**

Two days after the event of a right basal ganglia hemorrhage, a 78-year-old male reported a phantom arm protruding from his left shoulder. He could not see or touch the phantom arm but he felt the presence of an addition arm lateral to his paretic arm. Pain or sensory discomfort were absent in either the paretic arm or the phantom arm. He stated that he could intentionally move the phantom arm independent of his paretic arm. The examination showed that the passive movement of his paretic arm did not elicit any movement of his phantom arm. We diagnosed the SPL as a complication of the hypertensive basal ganglia hemorrhage and treated him with anti-hypertensive medications. His phantom arm persisted for 3 weeks, and it gradually faded away.

**Conclusion:**

SPL had been reported as a rare complication of various types of cerebral lesions. Right hemispheric lesions were most frequently associated with the SPL. Considering the intentional movement of the phantom arm, we deduced that the SPL might result from the impairment of the sensory feedback system for both internal body image and motor movement.

## Background

The phantom limb phenomenon is generally experienced by amputees who feel a persistent sensation of the limb that has been physically lost [1]. However, this phenomenon is also experienced by patients without amputated limbs. In such cases, this phenomenon is called a supernumerary phantom limb (SPL) [2]. The SPL had been reported as a rare complication of various neurologic disorders, including stroke, epilepsy, autoimmune disease, and trauma [2–7]. Herein, we report a case of SPL in a patient with a right basal ganglia hemorrhage and review the previous literature about this peculiar phenomenon.

## Case presentation

**Fig. 1:**
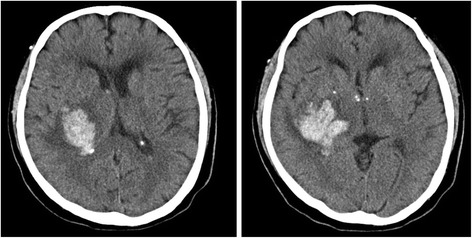
Axial brain computed tomography of the patient. The hemorrhage at the right basal ganglia extended medially to the thalamus and laterally to the external capsule

A 78-year-old male was admitted to our hospital with sudden onset headache and left-sided motor weakness. He was a well-educated retired engineer with a medical history of hypertension. A neurological examination revealed left-side homonymous hemianopia and motor weakness with the modified medical research council scale 2. There was a mild sensory impairment to light touch, pain, and temperature on the left face, arm, leg, and trunk. The proprioception was impaired in his left toe and finger. Tactile and auditory extinction were absent. Computed tomography (CT) of the patient’s brain showed a hemorrhage of the right basal ganglia extending medially to the thalamus and laterally to the external capsule with an approximate volume of 50 cm^3^ (Fig. 1). Considering his past medical history and the anatomical location of the hemorrhage, we diagnosed the hypertensive basal ganglia hemorrhage and treated him with anti-hypertensive medications.

**Fig. 2:**
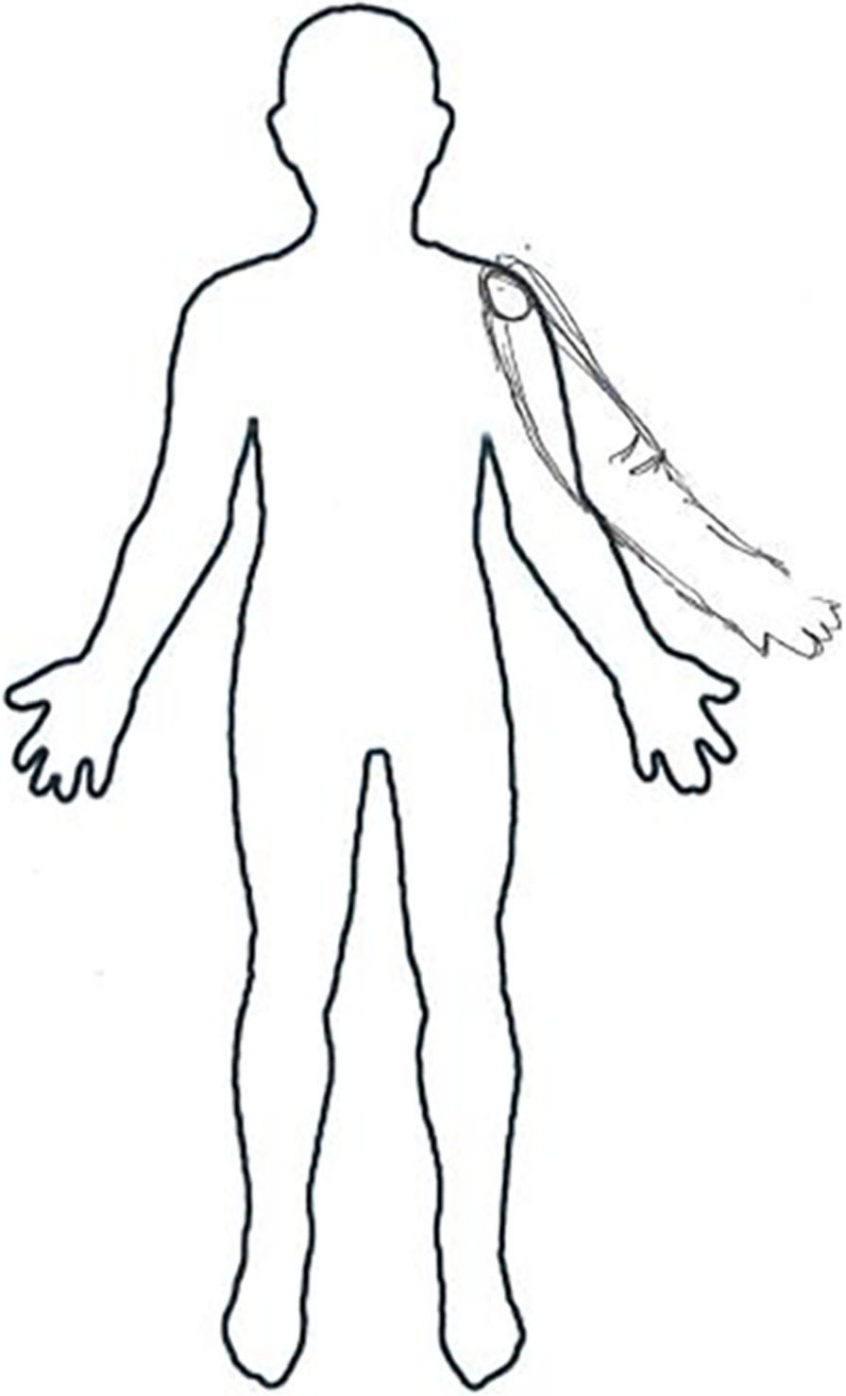
Drawing of the supernumerary phantom limb by the patient. The phantom arm protruded from the left shoulder

On the second day of hospitalization, the patient started to report the phantom arm. Beside his left paretic arm, he felt another arm protruding from his left shoulder. He could not see or touch this phantom arm, but he felt it very vividly. He reported that the phantom arm resembled his paretic arm in size and shape (Fig. 2). The phantom arm was not triggered by any specific condition; rather, it was persistently perceived. Closing his eyes or looking at his real arm did not influenced this false perception. Examination revealed that he could intentionally move his phantom arm. He stated that the movement was independent of his paretic arm and the strength was the same as his unaffected right arm. He could wave, grip or touch objects with his phantom hand. However, when we asked him to hold a ball with his phantom hand, he felt like the ball kept slipping out of his hands. Also, when we asked him to pretend using chopsticks with his phantom hand, he stated that it was clumsy because he was using the non-dominant hand. He also could not reach an object that was located far away from him with his phantom arm. He did not experience any involuntary movements of the phantom arm. When we put the glove on his paretic hand, we stated that he was wearing a globe on his paretic hand but not on his phantom hand. Passive movement of his paretic arm did not elicit any movement in the phantom arm. He did not complain of any pain or sensory discomfort in either the paretic arm or the phantom arm.

He was alert and oriented to time, place, and person with the Mini-Mental State Examination score of 30/30. He knew that having three arms is not normal and insisted that the phantom arm was not real. He showed no sign of seizure, and the electroencephalography showed that he had normal brain activity. A follow-up neurological examination revealed no deterioration, and brain CT showed no significant changes in the hemorrhage.

We diagnosed the SPL as a complication of the right basal ganglia hemorrhage. His phantom arm persisted for 3 weeks, and it gradually faded away.

## Discussion and conclusions

Through a literature review, we could find 28 well-described cases of SPL with the exclusion of cases not written in English, and without detailed descriptions and imaging diagnosis (Table 1).

**Table 1:** Clinical findings of the patients with the SPL

First Authors	Sex/Age	Site	Dx	SPL (number)	Onset time	Movement/Pain
Supraspinal lesion						
Lamb SC-K [24]	M/38	Lt. cerebrum	TBI, PTE	Arm (2) Leg (2)	7 weeks	+/−
Cipriani G [15]	M/75	Rt. TPC	ICH	Arm (1)	^a^	−/−
Millonig A [26]	M/70	Rt. TPC	Sz	Arm (1) Leg (1)	^a^	+/−
Yoo SD [27]	M/53	Lt. Pons	ICH	Arm (1) Leg (1)	2 months	−/+
	F/51	Lt. Pons	ICH	Arm (1)	2 months	−/+
Khateb A [19]	F/64	Rt. BG	ICH	Arm (1)	4 days	+/−
Srivastava A [28]	F/59	Rt. BG	ICH	Arm (3–5) ^b^	4 days	+/−
Tanaka H [25]	F/47 M/55	Rt. Pons Rt. Pons	ICH ICH	Arm (1) Leg (1) Arm (1) Leg (1)	1 month 1 month	+/− +/−
Staub F [13]	F/70	Lt. BG	CI	Arm (1) Leg (1)	1 month	+/−
Bakheit A [29]	M/71	Rt. BG	ICH	Leg (1)	2 months	−/−
Miyazawa N [30]	M/42	Lt. BG	ICH	Arm (1) Leg (1)	3 days	−/+
McGonigle D [20]	F/42	Rt. FL	CI	Arm (1) Leg (1)	3 weeks	+/−
Grossi D [21]	M/60	Rt. TL	ICH	Arm (1)	^a^	+/−
Canavero S [11]	F/61	Rt. BG	ICH	Arm (1)	6 weeks	−/+
Hari R [22]	F/37	Rt. FL	SAH, CI	Arm (1) Leg (1)	3 weeks	−/^a^
Mazzoni M [31]	M/66	Rt. BG	CI	Arm (1)	8 days	+/−
Worthington A [32]	F/72	Rt. TPC	CI	Hand (1)	Immediate	^a^
Halligan P [33]	M/80	Rt. MCA territory	CI	Arm (1)	6 months	+/−
Halligan PW [34]	M/65	Rt. BG	ICH	Arm (1)	3 days	−/−
Spinal or infraspinal lesion						
Melinyshyn AN [5]	F/31	PN	AIDP	Arm (2) Leg (2)	54 days	^a^/^a^
	F/57	PN	AIDP	Arm (2) Leg (2)	72 days	+/+
	F/68	PN	AIDP	Arm (2) Leg (2)	46 days	+/+
Belgrade M [7]	M/58	PN	NS	Toe (1)	^a^	+/+
Katayama O [35]	M/22	SC (C2)	TM	Arm (2)	2 years	−/+
Choi JY [36]	M/43	SC (C6)	TM	Leg (2)	6 days	−/+
Curt A [23]	M/71	SC (C3)	TM	Arm (2)	7 days	+/+
Sakagami Y [6]	F/31	SC (C4)	SjS	Arm (1)	^a^	−/+

^a^It was not described in the literature

^b^The number of SPL fluctuated

The supraspinal lesions included the frontal lobe, temporo-parietal lobe, basal ganglia, thalamus, and pons. These lesions were either on the right or left, but more frequently, they were found on the right side. All of them were contralateral to the side of the phantom limb. Phantom pain or sensory discomfort was reported in four cases.

Peripheral nerve and spinal cord lesions also caused cases of SPL. In most of the cases, the SPLs appeared bilaterally, and they were all associated with phantom pain. Previous studies and the review of the cases in this study demonstrate that the SPLs caused by the spinal cord or peripheral nerve lesions differed qualitatively from those caused by supraspinal lesions in that they were more associated with pain or sensory discomfort [8]. As for the cases of supraspinal lesions, our patient did not complain any pain or sensory discomfort in his phantom arm. Phantom pain is associated with ectopic neuronal discharges from an injured nerve and the sensitized dorsal horn in the spinal cord [9]. Some studies have demonstrated that phantom pain could be relieved by local anesthesia in a number of patients [10]. Therefore, it can be speculated that supraspinal lesions are less associated with phantom pain due to the absence of pathologic changes on the spinal cord or the peripheral nerves.

The reason why the SPL is more frequently associated with the right side lesion than the left side is still unclear. The right hemisphere is mainly involved in maintaining the internal representation of the body [11, 12]. Previous studies have proposed that the basal ganglia-thalamo-cortical loops are associated with the occurrence of SPLs by demonstrating their hyperactivity through functional magnetic resonance imaging [13]. However, the cases of various cerebral lesions and left hemispheric lesions also suggest that information about the representation of the body is distributed across various areas of the brain.

The time between the onset of SPL and the causative event varied from days to months. The short onset time as in our case suggests a rapid plasticity of body shape in the brain. This was well demonstrated in the rubber hand illusion [14].When the subject watched a rubber hand being touched synchronously with his or her own hand, the subject felt the rubber hand just like his or her own hand.

The body schema is continually modified by the integration of various sensory inputs as well as motor outputs [15]. Deafferentation by the cerebral lesions may cause a perceptual mismatch between the internal representation of the body and the sensory inputs from the ascending tracts, resulting in the dissociated images of body [16]. For our patient, the basal ganglia hemorrhage extending to the thalamus might have interrupted the integration of ascending sensory inputs, resulting in the phantom limb, which was not modified by the sensory inputs.

**Fig. 3:**
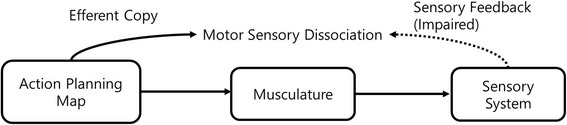
Proposed mechanism of the supernumerary phantom limb with its intentional movement. In absence of sensory feedback, the perception of the movement could be made based on its predicted outcome rather than on the actual outcome

One interesting finding was that our patient could control the movement of his phantom arm. Unlike his paretic arm, the strength of the phantom arm was the same as that of the normal arm. However, any sensory stimulation on the paretic arm did not elicit changes in the phantom arm. Therefore, we could infer that the phantom arm was influenced by the motor information but not by the sensory information. This can be explained by the efferent copy model [17]. In the intentional movement, the brain programs a set of muscle movements and uses a copy of the efferent motor command to match with the resulting movement [18]. In the absence of sensory feedback due to the cerebral lesion and the preserved efferent copy, the perception of a movement may be made based on its predicted movement rather than on the actual movement. This may cause a false perception of body movement (Fig. 3) [18].

Another interesting finding was that the patient could not use the chopstick with the phantom hand as skillful as with his dominant hand. This suggests that, the perception of the phantom arm might be based on the learned motor representation. Due to the lack of motor representation for using the chopstick, the patient could not use the chopstick with the phantom arm. Also, the patient’s phantom arm could not reach an object located far away. This suggests that, although his phantom arm was not influenced by sensory information, it was influenced by the preformed body representation.

Antoniello et al. demonstrated that among 50 post-stroke patients, 24 patients experienced the voluntary movement of their SPLs [8]. However, whether the SPL moved independently of the affected limb and whether the SPL was perceived only during voluntary action were not clearly described. Both voluntary and involuntary movement of SPLs were reported in many studies [13, 19–25]. In previous cases, it was reported that the SPL was triggered only during voluntary action [13, 19–21] or simply followed the action of the affected limb [22, 23]. However, our patient perceived his SPL without voluntary action and could move his phantom arm independently from his paretic arm. Through detailed examination of the SPL, we speculated that the SPL of our patient was caused by both the disintegration of sensory inputs on the preformed body image and the misperception of body image from the voluntary motor outputs.

In conclusion, our case as well as the reviewed cases illustrate that an SPL can be caused by various types of cerebral lesions, and it might result from the impairment of the sensory feedback system for both internal body image and motor movement.

## Abbreviations

AIDP: Acute inflammatory demyelinating polyradiculoneuropathy
BG: Basal ganglia
CI: Cerebral infarction
Dx: Diagnosis
FL: Frontal lobe
ICH: Intracerebral hemorrhage
Lt: Left
MCA: Middle cerebral artery
NS: Neurosarcoidosis
PN: Peripheral nerve
PTE: Post-traumatic epilepsy
Rt: Right
SAH: Subarachnoid hemorrhage
SC: Spinal cord
SjS: Sjogren’s syndrome
SPL: Supernumerary phantom limb
Sz: Seizure
TBI: Traumatic brain injury
TL: Temporal lobe
TM: Traumatic myelopathy
TPC: Temporo-parietal lobe
